# Psychological and reproductive decision-making experiences of young women after breast cancer diagnosis: a qualitative study

**DOI:** 10.1007/s00520-023-07880-7

**Published:** 2023-06-26

**Authors:** Jiajia Qiu, Lichen Tang, Ping Li, Guangyu Liu, Xinyi Rong

**Affiliations:** 1 Department of Nursing Administration, Shanghai Cancer Center, Fudan University, Shanghai, China; 2 Department of Oncology, Shanghai Medical College, Fudan University, Shanghai, China; 3 Department of Breast Surgery, Shanghai Cancer Center, Fudan University, Shanghai, China; 4 School of Nursing, Fudan University, Shanghai, China

**Keywords:** Breast cancer, Psychological, Reproductive decision-making, Experience, Young, Qualitative

## Abstract

**Background:**

Breast cancer has the highest incidence rate among malignant tumors in China, with a trend of affecting younger women. The treatment has short- and long-term adverse effects such as damage to the ovaries, which may result in infertility. Such consequences then increase patients’ concerns over future reproduction. At present, nor do medical staffs continuously assess their overall well-being, or ensure that they have the knowledge necessary to manage their reproductive concerns. This qualitative study aimed to explore psychological and reproductive decision-making experiences of young women who had experienced childbirth after their diagnosis.

**Methods:**

The phenomenological research, as a kind of qualitative study, was conducted on 12 young women who experienced childbirth after breast cancer diagnosis. Data collection was from September 2021 to January 2022 and content analysis method was used to analyze the data.

**Results:**

Five main themes were identified: (1) desire for childbearing from individual, familial, and social aspects after the diagnosis of breast cancer; (2) emotional experiences through pregnancy till raising children; (3) support needs from professionals, family, and peer; (4) self and doctors’ influencing factors on reproductive decision-making; and (5) satisfaction with the outcome of reproductive decision-making.

**Conclusions:**

The desire for childbearing of young women should be considered during the reproductive decision-making process. A multidisciplinary team is suggested to be set up to provide professional support. During the reproductive process, professional and peer support should be strengthened to improve decision-making abilities, alleviate negative emotional experience, and smoothen the process of reproductive experience for young patients.

## Background

Breast cancer has the highest incidence rate among all malignant tumor cases globally. In 2020, the number of new breast cancer cases globally reached 2.26 million, among which the number in China accounted for 420,000 in the same year [1]. Female breast cancer cases accounted for 19.9% of all malignant tumor cases [1], ranking first in the number of new cancer cases among women in China. The average age of breast cancer onset in China is 48.7 years, nearly 10 years earlier than that in Europe and the USA [2]. Developed by European School of Oncology-European Society for Medical Oncology (ESO-ESMO), the Fourth International Consensus Guidelines for Breast Cancer in Young Women (BCY4) defines young women with breast cancer as those diagnosed at age <40 [3]. Young women with breast cancer in China accounts for 10–15% of the entire breast cancer population [2, 4]. Data from the Shanghai Cancer Center, Fudan University, suggested that female patients aged 35 and younger accounted for 7.6% of all cases, and those aged between 35 and 44 accounted for 23.2% [5]. ASCO guidelines also called for attention to special issues including fertility in these young patients [6].

Ongoing developments in surgery, chemotherapy, radiotherapy, endocrine therapy, and other comprehensive treatments have improved the survival rate of young patients significantly. But these treatments have short- and long-term adverse effects on patients’ ability to conceive, particularly due to the ovarian injury. Furthermore, socio-economic progress, the implementation of a universal two-child and three-child policy, and late childbirth in China have meant that more breast cancer patients are unmarried or still have childbearing plans at diagnosis [7, 8]. Studies from China have suggested that about 35% of young patients with breast cancer plan to have children after diagnosis and treatment [9]. In China, the fertility management of patients with cancer primarily focuses on fertility preservation. Although the assisted reproductive technology increases the fertility rate in young patients indeed, only 14% of patients are aware of the technology, and only 2% have ever used it [10]. And currently, there is rarely a direct link between oncologists and fertility specialists in China, though sometimes they collaborate on the same multidisciplinary team.

The dilemma regarding childbirth that young women with breast cancer confront with is not just of medical nature; rather, it is also influenced by psychological factors [11]. Especially in traditional Chinese culture, childbearing is a responsibility that a woman should bear. After diagnosis, patients often have to make choices under multiple pressures within a short time, and most young patients are faced with difficult reproductive decision-making [12]. They are suffering from a huge psychological burden [13], lacking sufficient time to discuss fertility information [14], and yearning for professional guidance on fertility [15, 16]. Young patients are often unsure about their reproductive potential and are worried about their fertility status. Patients’ concerns about reproduction and raising children include worries about their reproductive ability and health, their children’s health, and childcare [17, 18]. Recently, fertility preservation in young patients with breast cancer has garnered increasing attention worldwide [19], but there is a lack of informative consultations, accurate patient education, continuous assessment, and mental health intervention to promote patients’ overall well-being. Even with mature fertility preservation measures, to what extent women’s concerns about fertility can grow is yet to be found out. Therefore, this study aimed to explore psychological and reproductive decision-making experiences of young women who had experienced childbirth after their diagnosis by using the phenomenological research and to provide a theoretical and practical scientific basis for future fertility management for this specific population.

## Methods

### Design and participants

The phenomenological research, as a kind of qualitative study, aims to describe people’s personal experiences, to understand people’s reactions to certain experiences by means of induction and description, and to capture some “real experience” [20]. Therefore, we chose and conducted the phenomenological research among young women with breast cancer who had experienced childbirth after diagnosis to understand their psychological and reproductive decision-making experiences. The method of this study and its reporting followed the Standards for reporting qualitative research [21]. This study was conducted in Shanghai Cancer Center, Fudan University, China.

Participants were approached individually when attending their medical follow-up and recruited by the corresponding author who is a master-degree nurse using purposive sampling. And all the participants were recommended by medical professionals who are familiar with patients’ childbirth status. The inclusion criteria were as follows: (1) females aged 18–40 years, with pathologically diagnosed breast cancer; and (2) had experienced childbirth after a cancer diagnosis, and voluntarily participated in this study. The exclusion criteria were as follows: (1) tumors in parts of the body other than the breast, and (2) verbal communication difficulties. The medical professionals finally recommended 15 participants who met the requirements. All the 15 young women with breast cancer who experienced childbirth after their diagnosis were approached; 12 of them took part in this study, and 3 patients declined because of time mismatch. The interview and data analysis were conducted simultaneously. When analyzing the data of the 12 interviewees, same themes repeatedly arose and no new themes arose. Therefore, the content analysis was believed to have reached saturation and the sample size could be determined.

### Ethical considerations

This study was approved by the Scientific and Ethical Committee of the Shanghai Cancer Center, Fudan University (IRB No:2 107239-15). And it was conducted in accordance with the Declaration of Helsinki (as revised in 2013). All methods were performed in accordance with the relevant guidelines and regulations. All participants signed informed consent forms, and they were informed that they could refuse to participate or withdraw from the study before the initiation of analysis, and they could refuse to answer any questions or stop the interview whenever they wanted.

### Data collection

Data were collected face-to-face using semi-structured interview guide from September 2021 to January 2022 by the corresponding author who is a master-degree nursing scholar with expertise on qualitative research. Written consents and demographic information were obtained from all participants prior to formal interviews. All interviews were conducted in a private and comfortable room and the audio was recorded.

The interview guide was developed based on the purpose of the study and literature review, and it was pilot tested with two patients. The interview questions included the following: (1) Can you talk about the intention to have children before and after the diagnosis? How did it change? (2) How did the intention occur to you? What factors contributed to the intention? (3) What was the process and outcome of reproductive decision-making? What were the influencing factors for your decision-making? (4) What were the needs and supportive sources in your reproductive decision-making? (5) How do you feel about the outcome of your reproductive decisions? (6) Is there any other fertility-related content you would like to share?

Additional questions were developed based on themes emerging from the first several interviews and were asked in subsequent interviews until data saturation was reached. For instance, when the theme “support needs from peer” emerged, we added additional questions such as “How did peer support work? What did you think about peer support?”

A total of 12 interviews were conducted. The length of the interviews was between 30 and 60 min. Field notes were taken for informal conversations during the interviews. The recordings were transcribed into text within 24 h after the interview.

### Data analysis

All recorded interviews were transcribed verbatim by the primary researcher. Data analysis was performed in conjunction with data collection. The content analysis method of Colaizzi [20] was used to analyze the data, which mainly includes seven steps: (1) repeatedly reading through the transcripts and getting familiar with them and taking initial notes; (2) extracting meaningful statements that are consistent with the phenomenon being studied; (3) generalizing and refining meaning from meaningful statements; (4) searching for common concepts or characteristics of meaning and form themes, theme groups, and categories; (5) relating the topic to the research phenomenon for a complete description; (6) stating the essential structure of the phenomenon; and (7) returning respondents to verify authenticity. NVivo software (version 11.0, QSR International, USA) was used to manage the data.

In this manuscript, all quotations from participants are in italics. Aliases (P1 to 12) were used for the participants.

### Trustworthiness of data

Several techniques were used to ensure the rigor of this study. Data saturation was strictly followed to guarantee the trustworthiness of the study. The interview and data analysis were conducted simultaneously. Content analysis was confirmed saturated and sample size was determined when no new themes arose. Two authors analyzed the data independently and compared their findings. All authors reviewed the themes and reached a consensus. Two participants were invited to discuss the content and to ensure that the findings were clear and understandable.

## Results

The mean (SD) age of the participants at diagnosis was 29.67 (SD=4.12) years. Four were single and eight were married at cancer diagnosis. Besides two participants with junior college certificate, all the others had either a bachelor’s degree (*n* = 7) or a master’s degree (*n* = 3). Only one participant was primiparous before their cancer diagnosis. All participants underwent surgery, 8 received chemotherapy, 3 received neoadjuvant chemotherapy, 7 received radiotherapy, 9 received endocrine therapy, and 3 received targeted therapy. Two of them adopted assisted reproductive technology. The range of time from diagnosis to birth-giving varied from 13 to 68 months (mean 47.17 months). Table 1 shows the demographic characteristics.

**Table 1:** Demographic characteristics of participants (*n* = 12)

No.	Age at diagnosis (year)	Marital status at diagnosis	Education	Treatment and stage of disease	Desire for childbearing before diagnosis	Desire for childbearing after diagnosis	Gestational age (year)	Abortions prior to the pregnancy	Assisted reproductive technology	Time from diagnosis to birth-giving (month)	Single pregnancy or twin pregnancies	Lactation
P1	28	Married, childless	Junior college	Surgery+chemotherapy+endocrine therapy (stage IIA)	No	Yes	33	No	No	68	Single	No
P2	29	Married, childless	Master	Surgery+chemotherapy+radiotherapy+targeted therapy+endocrine therapy (stage IIA)	Yes	No	32	No	No	50	Single	No
P3	30	Unmarried, childless	Master	Surgery+chemotherapy+radiotherapy+endocrine therapy (stage IIA)	No	Yes	31	No	No	13	Single	No
P4	40	Married, childless	Bachelor	Surgery+endocrine therapy (stage IIA)	Yes	Yes	44	Yes	Yes	66	Single	No
P5	27	Married, childless	Bachelor	Neoadjuvant chemotherapy+surgery+chemotherapy +radiotherapy+targeted therapy+endocrine therapy (stage IIIA)	No	No	31	No	No	56	Single	No
P6	33	Married, childless	Master	Surgery+chemotherapy+endocrine therapy (stage IIA)	Yes	Yes	36	No	No	45	Single	No
P7	30	Married, with 1 child	Bachelor	Surgery+chemotherapy+radiotherapy+endocrine therapy (stage I)	Yes	No	33	No	Yes	48	Single	No
P8	27	Unmarried, childless	Bachelor	Surgery+endocrine therapy (stage I)	No	No	31	No	No	56	Single	No
P9	24	Unmarried, childless	Bachelor	Neoadjuvant chemotherapy+surgery+radiotherapy+targeted therapy (stage IIA)	No	No	27	No	No	41	Single	No
P10	30	Unmarried, childless	Bachelor	Surgery+chemotherapy+endocrine therapy (stage I)	Yes	No	34	No	No	60	Single	No
P11	26	Married, childless	Bachelor	Neoadjuvant chemotherapy+surgery+radiotherapy (stage IIB)	No	No	29	No	No	44	Single	No
P12	32	Married, childless	Junior college	Surgery+chemotherapy+radiotherapy (stage I)	No	Yes	33	No	No	19	Single	No

Overall, five main themes were identified: (1) desire for childbearing from individual, familial, and social aspects after the diagnosis of breast cancer; (2) emotional experiences through pregnancy till raising children; (3) support needs from professionals, family, and peer; (4) self and doctors’ influencing factors on reproductive decision-making; and (5) satisfaction with the outcome of reproductive decision-making.

**Fig. 1:**
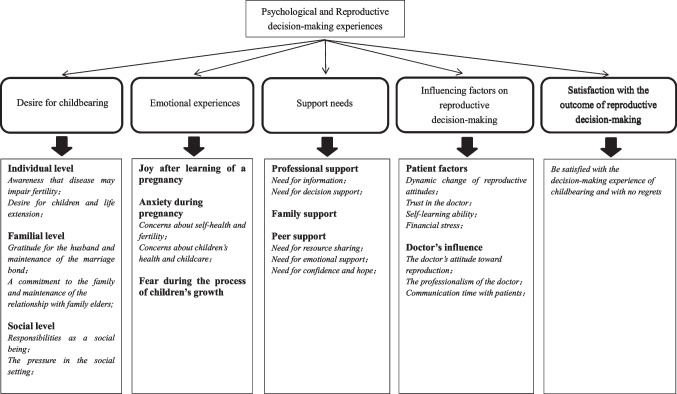
A summary of themes, sub-themes, and key codes developed in this study

Detailed reports on the five themes with supportive descriptive exemplars are shown in Fig. 1. All quotations from participants are in italics. Aliases (P1 to 12) were used for the participants.

## Desire for childbearing from individual, familial, and social aspects after the diagnosis of breast cancer

### Individual level

#### Awareness that disease may impair fertility

Under normal circumstances, many young couples think that achieving pregnancy is assured. But they will change their mind when they realize that their fertility may be affected. *Because I know chemotherapy does great damage to the ovaries, destroying its function, which is a gift for women. When mine is deprived, I will never regain it. One doesn’t treasure things till she has lost them...So I really want to have a child (P12, 32 years old).* The diagnosis of breast cancer even passively drives a patient’s desire for childbearing to some extent.

#### Desire for children and continuation of life

Some young patients decide to have children because of their fondness for children and will experience regret if they do not have any. *I like children, and I will feel sorry that I can’t be a mother with a child* (*P8, 27 years old*). Some patients think reproduction is a goal they must accomplish in life, as well as a continuation of life. *You get married, then you have offspring. If you don’t have any children, you feel like your life goal is not accomplished. This idea could haunt in your mind (P4, 40 years old).*

### Familial level

#### Gratitude for the husband and maintenance of marriage bond

While feeling supported by their husbands, young women also detect their husbands’ innermost, desire, and want to express gratitude through childbearing. *On our way to chemo, while he was driving, he saw children, and he smiled. I could feel that he liked children indeed…I know the man is reliable although there is still a long way to go...I made the decision to have children for my husband (P3, 30 years old). When I told him I was ill, he told me not to worry, and he would not abandon me... He’s been so supportive and helpful to me. I don’t want my husband to be lonely, so I want to leave him with a child (P11, 26 years old).*

#### A commitment to the family and maintenance of the relationship with family elders

Some young patients may hope to relieve the reproductive pressure from their family elders by giving birth. *Behind the marriage, there are family elders...Especially as my husband is the only child, we confront a lot of pressure from his parents who wanted us to have children. My husband supported my decision to be DINK. In fact, it would be fine for us to live a lifetime on our own, but the pressure from my husband’s family is very great (P10, 30 years old).*

### Social level

#### Responsibilities as a social being

As social beings, women have been given the social responsibility of bearing children since ancient times, and young women with breast cancer are no exception. *To have children is to accomplish the life goal, because I think this is the social mission that a woman should fulfill (P4, 40 years old)*.

#### The pressure in the social setting

As a social being, one is always affected by the external environment of society. Some young patients say that they are jealous when they see the growth of the children of their peers and colleagues. *I feel lonely, and my soul has nowhere to reside...If I had a child and I could be called mother, I think the happiness would be extremely strong......We can’t live like a single cell. In fact, I am eager to have children and have a perfect family atmosphere like others (P12, 32 years old).*

## Emotional experiences through pregnancy till raising children

### Joy after learning of pregnancy

The incredible joy after learning of pregnancy helps young women better return to normal life after experiencing diagnostic shock and intensive treatment. *It’s really a matter of destiny...when the pregnancy test stick showed positive, I hurried to the hospital to check. I was pretty happy when verified as pregnant (P10, 30 years old).*

### Anxiety during pregnancy

#### Concerns about self-health and fertility

Experiencing breast cancer is a stressful event for patients. In balancing disease and fertility, young women are still full of anxiety and fear of recurrence and metastasis. *I was always wondering if childbearing would cause recurrence and metastasis. The fear persisted just like hanging the sword of Damocles over my head. From the moment I decided to have a baby, the sword kept hanging over my head because it felt like I was driven to the last ditch (P10, 30 years old).*

#### Concerns about children’s health and childcare

In addition to worrying about their own health, young women often worry that breast cancer treatment will affect the health of their unborn child. *I couldn’t sleep the night before I had a prenatal examination...I am worried about the baby. I hope it is healthy. I read <Heart Sutras> to calm myself down (P4, 40 years old)*. The genetic predisposition to breast cancer also raises concerns about potential genetic risks for children. *I have a daughter, but I would rather I had a son, just because I didn’t want her to risk in childbearing. The other fear is genetic cancer. I am afraid that she might get breast or uterine diseases (P3, 30 years old).*

### Fear during the process of children’s growth

More fear occurs during children’s growth because of gender. *I’m just a little scared...... I have a daughter, and I pay close attention to her growth. I am afraid that my hormone level will be passed on to her, giving her high-risk of breast cancer...In addition, as she grows up, it becomes inconvenient for me to take her to a changing room when we go swimming or bathing together (P1, 28 years old).* Being unable to bring up children because of shortened lifespan also worries young patients. *Every time I come to the cancer hospital for examination, I am still quite afraid. In fact, after having experienced so much, I don’t care much about my physical health. The reason why I am afraid of death is that my daughter is still young, and I fear that she will be left with a stepmother after I pass away. (P3, 30 years old).*

## Support needs from professionals, family, and peers

### Professional support

#### Need for information

Some patients lack information support. They do not realize the impact of breast cancer and related treatment on fertility and fail to implement fertility protection measures in time. *I didn’t think that (breast cancer and related treatment) would affect reproduction... I probably had heard about the ovary-protecting injection in the process of chemotherapy, but it was before I learned this (breast cancer) and related treatment might affect fertility. I asked the doctor, and he said there was no need to do so as the treatment had already started. I had to skip the injection and continued the treatment to the end, according to the original process (P9, 24 years old).* Some patients obtained post-breast cancer fertility-related information through medical staff, fellow patients, the internet, books, and literature, but the available information was also relatively limited. *Is there any risk? Is the risk manageable? What is the exact time of birth? Besides the above information, after giving birth, I also specially consulted the doctor about breastfeed, reviews during childbearing, and when I should restore treatment after birth. As a matter of fact, these things I care about all have much to do with myself and my children (P6, 33 years old).*

In this study, most of the patients expressed willingness to further understand the issues, such as fertility risk, the timing of childbearing, reexamination during childbearing, postpartum breast milk, and when to resume treatment after childbirth.

#### Need for decision support

Some young patients have a relatively clear desire for parenthood when they are faced with reproductive decision-making, but they are more dependent on the judgment and support of doctors when making such decisions. *Actually, asking my husband for his advice on childbearing is just like asking for his advice on shopping. In fact, I have already made the decision in my heart. I just want to canvass, get someone on my side. I heard that Professor L is very supportive of having children, so I want to see him (P7, 30 years old).* In addition, the fertility of young breast cancer patients is a complex issue, and some patients hope to receive standardized guidance and comprehensive decision support. *No one, not even the cancer hospital, can give me instructions on what checks I should do and how to do, and so on throughout my pregnancy...Because there are a lot of women about my age who are sick, and most of them have the desire for childbearing, but there is no standardized guidance (P11, 26 years old).*

### Familial support

Under the influence of multiple pressures, young patients are eager to receive emotional support from others, especially the understanding and support from family members, among which the support from partners is the greatest comfort. *First of all, my husband is just like my comrade-in-arms. He must be absolutely willing to communicate with me unconditionally and speak from the heart if he has any thoughts. This is exactly what I hope my comrade to do. In this way, I am willing to actively cooperate with him to do whatever I can (P10, 30 years old).* Some couples choose to discuss to sort through problems and support the patient. *Both of us were lost at the beginning, but my husband was calmer than me. He discussed with me on whatever we could think of such as what results we might have and what state we might be in in the future...I think discussions are very important (P5, 27 years old).*

Of course, support from other family members, including parents, is also important in making reproductive decisions. *I feel that my mother has given me a second life. She takes care of my daily life meticulously...My parents gave me so much support, which kindled my desire to live and go on (P12, 32 years old).*

### Peer support

#### Need for resource sharing

The sharing of information and resources among patients helped to remind each other of their fertility options. *Peer patients can refer to my case when deciding to have children. On one hand, I’m encouraging them. On the other hand, I’m giving them a reminder of the problems they might face during their pregnancy. (P10, 30 years old).* Information from similar cases could be of reference for young women. *I once attended a lecture on reproduction. A successful mother shared with us the whole process, including how to make decisions and how to take care of children. Her case cleared the way for us and strengthened our confidence, making us believe that having children is possible. The sharing of successful cases is very important because it is the first step to establish more communication (P5, 27 years old).*

#### Need for emotional support

The young patients in the study all mentioned the positive inspiration drawn from other cases and the need for interaction and mutual assistance among patients. *When I was in the hospital, a nurse set up a group of young patients for the preparation of her graduation thesis, and I found it very helpful for me. Young patients especially need peer support because they seldom meet other patients about their own age... When she meets a young patient, she feels like she has really met someone who understands her. The cheerful and humorous conversation really helps (P2, 29 years old).*

#### Need for confidence and hope

Sharing successful cases between patients enhanced the confidence of young patients and ignited their hope for reproduction and life. *When we were just diagnosed, we were at a loss. We asked for help everywhere...Patients in the communication group usually had empathy and could think from the perspective of others. I joined in a group called Waiting Angels, and all the group members had not got children yet. When someone had a baby, the good news would be shared in the group. We would congratulate and celebrate, and we would become more confident (P8, 27 years old). I gave a lecture to the patients, and I met them with my children in person. This is the best way because we are living proof. The doctors and nurses may not understand this feeling, nor the healthy people. I showed my physical condition (triple-negative breast cancer). It’s been already 8 years since my treatment, and my child has been 4 years old. Look at me! How could you have no confidence in yourselves? (P11, 26 years old).*

## Self and doctors’ influencing factors on reproductive decision-making

### Patient factors

#### Dynamic change of reproductive attitudes

For young patients, reproductive attitudes could change dynamically with the changing personal, familial, and social environments. Some patients prioritized their life and health after diagnosis and choose to postpone their reproductive plans. However, as they recovered, their desire for reproduction gradually increased. *One or two years after diagnosis, I really put the survival in the first place of my life. I didn’t think much about anything else at that time. After chemotherapy, I lost all my hair and my confidence too. I didn’t think about my future. I could only go one step further and look ahead. Three or four years after the disease stabilized, I gradually resumed work and my life returned to the normal track (P1, 28 years old).* Some patients did not have the desire to have children before diagnosis. But when they were aware that they might lose the opportunity to have children due to the loss of fertility, the desire awaked and became even more urgent. *I used to be a dink. I didn’t want to have children. I thought children were so annoying...Later, I reflected on myself. The real reason I didn’t want to have children was that I thought I was pretty and wanted to keep in good shape. I was not a real dink, and I was just too young to get myself ready to be a mother. In fact, this illness had a good side as it awakened my desire for childbearing and left me no choice. It made me realize that life should be lived in a different way (P12, 32 years old).*

#### Trust in the doctor

In the study, patients’ trust on doctors and science strongly influenced their reproductive decisions. *I trust in Doctor L because of his expertise. I believe in him. I also have trust in the doctors of National Hospital for Women and Infants. I feel very confident with the support of the two well-known hospitals in China (P9, 24 years old). I totally trusted Professor H at that time. I knew nothing and sometimes I was confused, but the doctor was patient. He was very important to me and my whole family (P10, 30 years old).*

#### Self-learning ability

The interviewees in this study were young patients under 40 years old who had certain self-learning abilities and believed in science through self-directed education. *I will consult relevant materials to relieve my nerves. For example, I will read the relevant materials from the annual medical summit, because I think that only with strong theoretical knowledge reserve can I have a solid foundation to relieve my tensive mental state. There are few domestic cases, among which I don’t believe those against having children or having sex...Before I go to professor Y, I usually list everything I need to ask on a piece of paper, and he will analyze it for me according to my needs (P3, 30 years old).*

#### Financial stress

Finance is the material basis for reproduction. The cost of medical treatment, assisted reproductive technology, and raising children makes patients and their family think twice about having children. *My husband used to look forward to having a child, but when I was ready to stop taking the medicine, he suddenly backed out. We had a quarrel about it...Later, I realized that he was thinking about the financial stress brought by having children (P2, 29 years old).*

### Doctor’s influence

#### The doctor’s attitude toward reproduction

Doctors’ attitudes may greatly influence the reproductive decisions of young patients. If the doctor pays attention to whether the patient has reproduction needs and proactively mentions reproduction issues during the diagnosis and treatment process, the patient will focus and consider this issue earlier, rather than feeling regret after losing the function. *I was ready for chemo, then the doctor directly told me that I needed an injection to protect my ovaries. He elaborated on its purpose. Perhaps I didn’t plan to have children in the future, but he was responsible to help me keep the function. Maybe in 2 or 3 years, when the idea occurred to me, I was still able to give birth (P2, 29 years old).* The relaxing attitude of the doctor can also make the patients feel comfortable, so they can better tackle their fertility concerns. *Professor H defended me against my mother-in-law who thought I was unable to have children after operation...And later when I went to the check-up, he brought it up to me and asked whether I wanted to have a baby. If yes, he told me I could stop taking pills and to prepare for pregnancy...Dr. H’s attitude was so relieving that it made me feel natural to have children (P10, 30 years old).*

#### The professionalism of the doctor

Doctors’ attention to the reproduction issues of young patients and the provision of professional information is an excellent motivator for patients to seek medical treatment and have children. *When it comes to making such an important decision, I think I will feel more confident in finding a prominent doctor whose professional information will make me more confident... I don’t worry when I am certain. The uncertainty of information may exacerbate the fear (P9, 24 years old).* In addition to professional information, medical support can also greatly encourage young patients to make reproductive decisions and gain mental strength. *I think both doctors and nurses can give patients spiritual support and hope. Because no matter how strong the cancer patient is on the outside, you can’t see how fragile she is on the inside. Doctors and nurses were lifesavers to the patients who were once very close to death (P12, 32 years old).*

#### Communication time with patients

When young patients make reproductive decisions, to make themselves feel more confident and secure, they hope to discuss and consult with professionals, even experts in this field, to achieve their desired results. However, medical resources are limited, especially in big hospitals in first-tier cities. Doctors face so many patients from all over the country every day that they tend to pay more attention to the treatment of diseases and the continuation of life and health instead of assisting them on reproductive decision-making. *For example, I wanted to see Doctor L when I intended to make reproductive decisions. But I was unable to register because there were too many patients waiting in the queue...However, when it comes to making such important decisions, I will feel more confident if a prominent doctor can give me advice (P1, 28 years old).*

## Satisfaction with the outcome of reproductive decision-making

In this study, all interviewees expressed satisfaction with the experience of reproductive decision-making, and there were no regrets. After enduring breast cancer and going through the process of childbearing, the patients were satisfied with their choices. *I have no regrets about my decision to have children...I think the thing that makes a man grow up quickly is to have children. I want to have a common purpose for us. That’s how I feel about life (P3, 30 years old). No regrets at all, though challenges appear. I have a child, I fight for her and I upgrade, like playing RPG games (role-playing games) (laughter) (P6, 33 years old).* Some patients even plan to continue having children after their first birth. *My son has been almost 3 years old, and I have no regret...I am thinking about having a second child. I originally wanted a daughter when I was pregnant (P9, 24 years old).* One of the patients regretted the timing of childbearing in the process of the reproductive decision, but she still agreed to the decision. *Actually, I favored it (the thoughts and actions then). I just regret if I should have had children earlier, but I was afraid of instability and recurrence of the disease (P8, 27 years old).*

## Discussion

### Attention should be paid to young patients’ desire for childbearing and its changes

The participants in this study were all young women who had experienced childbirth after breast cancer. The results showed that their desire for childbearing changed with marital status, disease diagnosis and treatment, and rehabilitation degree, which was also supported by previous studies [22, 23]. After diagnosis, patients focused more on the treatment of the disease, the continuation of life, body recovery, and other aspects; reproduction issues were not the priority. With the completion of treatment, the threat to life gradually decreased. In addition to the influence of family, society, and other aspects, the patients’ reproductive-related issues increased prominently. However, in the clinic, discussion of reproductive decision-making issues was lacking, and the needs for reproductive protection were not fully met [9, 24, 25].

Therefore, medical professionals should pay attention to their patients’ desire for childbearing and its dynamic changes as soon as possible and strengthen their attention to the reproduction of young patients. The discussion of reproduction should run through the whole process of patients’ diagnosis, treatment, and rehabilitation. The medical team consisting of oncologists and fertility specialists could give advice on fertility preservation and future childbearing. Meanwhile, a multidisciplinary team including psychologists and nurse specialists should be established to encourage young women to express their thoughts and confusion about reproduction so that their hidden needs can be identified and targeted support can be provided. Based on the dynamic trend of reproductive intention, reproductive decision-making issues can be discussed with patients in different stages and aspects. At the initial stage of diagnosis and treatment, the formulation of the diagnosis and treatment plan, and the different stages of disease rehabilitation, medical professionals should detect whether the patients have reproductive needs and whether they have changed their reproductive intentions. At the initial stage of diagnosis, the discussion could focus on reproductive protection. The patients can realize the importance of reproductive protection and can reserve enough time to implement reproductive protection measures. Before and after the formulation of the diagnosis and treatment plan, the patient’s reproductive issues should be fully considered, and the corresponding diagnosis and treatment plan should be formulated and adjusted based on the discussion results with the patients. Effective contraceptive and family planning guidance should be given to patients who temporarily have no reproductive intention to avoid unintended pregnancy. In the rehabilitation process, the focus should gradually shift from treatment to reproduction, providing information and decision-making support for post-cancer reproduction, including genetic testing, artificial insemination, pregnancy review, and other related content, to improve the efficiency and quality of reproduction care and services.

### Attention should be paid to the emotional experience of young patients in the reproductive decision-making process

In this study, the young patients who experienced childbearing often had very firm beliefs about reproduction in the decision-making process, but the young women still experienced many positive and negative ambivalent emotions during the whole decision-making process. There are many contradictions in the decision-making process: ignoring the impact of disease on fertility but worrying about losing fertility; desire for parenthood, but being afraid of the recurrence and metastasis of the disease; longing for a daughter, but worrying about the inheritance of disease or cancer predisposition; and enjoying the success of pregnancy, but worrying about not being able to accompany the child in the future. This is consistent with the results of Ghaemi et al [26] and Faccio et al [27]. Currently, the emotions related to reproduction have received increasing attention from Chinese and international researchers [28, 29]. Young women have different degrees of worry and fear about their fertility, health, children’s health, care for children, and other aspects in the process of reproductive decision-making. In the professional field, more attention is paid to the preservation of fertility in young patients with breast cancer. Despite the mature fertility protection measures, the pressure of childbearing after diagnosis and treatment of cancer may be too much for the young patients to bear, causing their concerns about reproduction issues to be more durable than the psychological pressure incurred from the tumor. Childbearing is the only source of human reproduction. Traditional Chinese concepts believe that women bear the mission of childbirth and carrying on the family line, so reproduction is a personal issue, a family demand, and a reflection of social responsibilities entrusted to women [30]. Participants in our study were all inclined to be influenced by this concept. Therefore, helping young patients to correctly understand the disease and reducing their adverse moods should be the focus of the professional medical staff. In different stages of cancer diagnosis, treatment, and rehabilitation, patients’ different reproductive needs and changes in their psychological state should be continuously evaluated. Individualized and precise support should be given to patients to relieve their anxiety, meet their emotional needs, and realize social value [31].

### Establish a support system of professional reproductive information for young patients to improve their decision-making ability

Both Chinese and international guidelines strongly suggest that patients’ reproductive intentions should be identified after cancer diagnosis and before the formulation of a treatment plan [3, 19]. Reproduction-related functions should be evaluated, and professional reproductive counseling should be provided. Young breast cancer patients are faced with unique physiological, psychological, and social problems, so the diagnosis and treatment plan and reproductive management plan should be refined through multidisciplinary consultation and interdisciplinary discussion to finally formulate the treatment and fertility preservation plan [32]. Relevant studies indicate that receiving multidisciplinary expert consultation, especially fertility expert consultation, is beneficial for patients to make reproductive decisions and alleviate their reproductive concerns [33]. A more in-depth fertility consultation can lower the anxiety level of young patients [34]. We suggest setting up an interdisciplinary team to provide professional support for young patients in the form of multidisciplinary cooperation that can build effective communication channels, coordinate personnel and task allocation, and accomplish the follow-up and communication of young patients’ reproduction. This will help break the theoretical and practical barriers and encourage young patients to make better reproductive decisions.

Since medical staff are the main medical decision-making influencers in China, they play an important role when patients lack technical knowledge [35, 36]. Therefore, medical staff should strengthen communication with young patients. Respondents in this study also said the doctors’ mention of reproductive issues helped them realize that fertility needed to be considered even as they were experiencing the shock of diagnosis, and the doctors’ relatively relaxed attitude and professional support also gave them great encouragement and confidence. The better the rapport between patients and doctors, the more likely their attitudes toward preservation of reproductive ability can be coordinated. Studies show that reproductive education plays a positive role in improving disease-related knowledge and relieving reproductive concerns [37]. Doctors with a stronger sense of responsibility are more willing to communicate with patients and provide effective consultation [38].

During the process of providing professional support for patients, some decision-making aids for patients can be appropriately introduced to facilitate better informed decisions. Decision aid tools provide more targeted information, more detailed content, and more visualization charts to present data for patients to understand in a more intuitive way the “possibility” evidence in scientific study. These tools help patients think about the benefits, risks, and uncertainties of various options and improve their readiness and quality of decision-making [22]. They have been used in Australia, Portugal, the Netherlands, Canada, and other countries [39–42]. In Taiwan, Tseng et al. [43] constructed a web-based decision-making aid tool through action research. They applied it to breast cancer patients of childbearing age to help them make assisted decisions on fertility preservation, achieving good results. Therefore, it is necessary to establish a reproductive information support system for young patients, to develop suitable reproductive decision-making aids for Chinese local conditions, and to provide evidence-based information support to reduce decision-making conflicts and reproductive concerns of patients.

### Strengthen peer support, give confidence, and hope

The results of this study show that peer support plays an important role in the reproductive decision-making process of young breast cancer patients. Sharing this direct experience makes the patients who want to have children realize their reproductive possibility, correct wrong perceptions, build confidence, and generate hopes. Peer support is a kind of social psychological support that provides social, emotional, and information support for target groups by sharing experience, emotion, information, and ideas with people of similar age, behavior, life experience, and cultural background to realize disease prevention and health promotion. It can improve breast cancer patients’ mental, physical, social, and cognitive functions; make them feel a sense of belonging and reduce loneliness; and ameliorate their overall quality of life [44]. Existing research shows that the degree of reproductive concerns of breast cancer patients of reproductive age has a “delayed” trend, and patients in the recovery stage may have more anxiety [45]. The successful experiences of the patients who have already given birth are most convincing. Patients can obtain emotional and information support through communication and mutual assistance between peer patients. The successful experience of peers can encourage patients, and the satisfied reproductive decision-making experience can also serve as a good example of peer support, forming a positive cycle.

In clinical practice, provision of professional information plus peer support provides comprehensive support for patients. In our center, peer volunteers were usually trained before they offer peer support. In the training, they learned professional information and communication skill and learned to provide positive support and avoid negative emotions. Upon the completion of the training, peer volunteers would provide one-on-one interview for the young patients in need with the monitoring of professionals in the ward on a weekly basis. All the participants in this study were satisfied with the outcome of their reproductive decisions and never experienced regret.

Peer support has unique advantages over other forms of social support [46]. Because of similar experiences, peer supporters can better understand patients’ feelings and provide corresponding emotional support through empathy. Patients are also more willing to express their thoughts and feelings to peer supporters and expose their inner vulnerability. At the same time, peer supporters can reduce others’ uncertainty by sharing their own experiences, and information verified by peer supporters is more easily accepted and recognized by patients. Of course, the peer support model also has obstacles such as inconvenient transportation, patients’ cognitive deficiency, negative emotions, and physical symptoms. The interviewees in this study also indicated that the communication between patients and the answers to questions sometimes made them more anxious. Some patients also felt pressure when they saw their fellow patients get pregnant and felt as though they “lived in someone’s shadow.”

We suggest further enriching the forms of peer support in clinical practice, strengthening the publicity of peer support, making full use of social network platforms, widening the ways for patients to participate in peer support, breaking the time and space obstacles of peer support, and enhancing patients’ understanding of peer support. At the same time, it is necessary to provide professional training for peer supporters, focus on the mental health status of peer supporters, and provide relief and help. During peer support, it is better to accurately match supporters and patients for better homogeneity between supporters and patients in terms of demographic characteristics, disease condition, and reproductive attitude to achieve a better peer support effect [47].

## Limitations and suggestions

The subjects of this study were mainly young women with breast cancer treated in the Department of Breast Surgery of Fudan University, Shanghai Cancer Center. The sample size was small, and the location was limited in Shanghai, excluding the young patients living in other regions and treated in other hospitals. The representation and promotion of the sample are insufficient. And we didn’t compare participants with non-oncological sample and those young women who haven’t experienced childbirth after diagnosis. The analysis based on the type of therapies and performed treatment also lacked. The discussion was all about having hope and achieving pregnancy, and the consideration of those who might not achieve a desired pregnancy was also lack. We could divide our participants into diverse samples in the future (e.g., divide women into those who received neoadjuvant chemotherapy and those who did not, divide women into those who were still undergoing treatment when they conceived and those who gave birth after the conclusion of treatment, divide women into those who had and had not achieved a desired pregnancy).

It is recommended to conduct continuous follow-up of respondents to further understand the long-term impact of reproductive decision-making on patients and their long-term experience and to construct a reproductive decision-making support program and execute a rigorous-designed intervention study, based on young patients’ reproductive experience. It is also suggested that a multidisciplinary team consisting of oncologists, fertility specialists, psychologists, and nurse specialists be set up to provide professional support for young patients.

## Conclusion

Through interviews with young women with breast cancer who have made reproductive decisions, we found that the reproductive decision-making process of young women with breast cancer was affected by many factors. Therefore, the desire for childbearing of young women with breast cancer should be considered during the reproductive decision-making process. A multidisciplinary team consisting of oncologists, fertility specialists, psychologists, and nurse specialists is suggested to be set up to provide professional support. During the reproductive process, professional and peer support should be strengthened to build effective communication channels for reproductive decision-making, improve decision-making abilities and experience, alleviate negative emotional experience, and smoothen the process of reproductive experience for young patients.

## Data Availability

The datasets used and/or analyzed during the current study are available from the corresponding author on reasonable request.
